# Windblown Pliocene diatoms and East Antarctic Ice Sheet retreat

**DOI:** 10.1038/ncomms12957

**Published:** 2016-09-20

**Authors:** Reed P. Scherer, Robert M. DeConto, David Pollard, Richard B. Alley

**Affiliations:** 1 Department of Geology and Environmental Geosciences, Northern Illinois University, DeKalb, Illinois 60115, USA; 2 Department of Geosciences, University of Massachusetts, Amherst, Massachusetts 01003, USA; 3 Earth and Environmental Systems Institute, Pennsylvania State University, University Park, Pennsylvania 16802, USA

## Abstract

Marine diatoms in tillites along the Transantarctic Mountains (TAMs) have been used to suggest a diminished East Antarctic Ice Sheet (EAIS) during Pliocene warm periods. Updated ice-sheet modelling shows significant Pliocene EAIS retreat, creating marine embayments into the Wilkes and Aurora basins that were conducive to high diatom productivity and rapid accumulation of diatomaceous sediments. Here we show that subsequent isostatic uplift exposed accumulated unconsolidated marine deposits to wind erosion. We report new atmospheric modelling utilizing Pliocene climate and derived Antarctic landscapes indicating that prevailing mid-altitude winds transported diatoms towards the TAMs, dominantly from extensive emerged coastal deposits of the Aurora Basin. This result unifies leading ideas from competing sides of a contentious debate about the origin of the diatoms in the TAMs and their link to EAIS history, supporting the view that parts of the EAIS are vulnerable to relatively modest warming, with possible implications for future sea-level rise.

## Abstract (web summary)

A long-standing debate regarding the Pliocene history of the East Antarctic Ice Sheet was spurred by the discovery of marine diatoms in the Transantarctic Mountains. Here the authors show that the diatoms were emplaced by wind following a retreat of the ice sheet into coastal basins and subsequent isostatic emergence.

## Graphical abstract

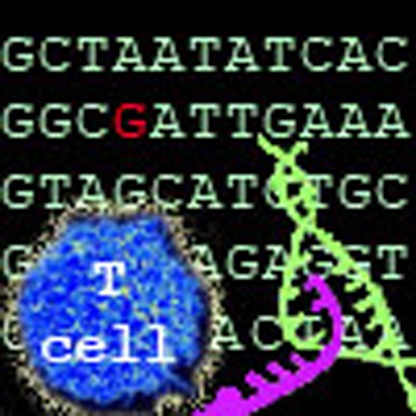

The susceptibility of the marine sectors of the West Antarctic Ice Sheet to retreat^1^ and its history of past collapse, are now well documented^2^,^3^,^4^, whereas uncertainty with regard to the history^5^,^6^,^7^,^8^ and stability^9^ of parts of the East Antarctic Ice Sheet (EAIS) during Pliocene warm intervals continues. Past EAIS variability has been a matter of debate and speculation since the publication of a series of strongly contrasting papers that followed a controversial report by Webb *et al*.^10^ that inferred major EAIS retreat along the Wilkes, Aurora and Pensacola subglacial basins of East Antarctica during early- and mid-Pliocene warm intervals, based on marine diatoms extracted from tillites in the Transantarctic Mountains (TAMs). These tillite units are collectively referred to as the Sirius Group, or Sirius Formation in older literature.

Strongly divergent interpretations developed of either long-term ice-sheet stability^11^,^12^,^13^,^14^ or ice-sheet dynamism through the Pliocene^15^,^16^,^17^, based on analysis of sediments from the TAMs, yet none of the dozens of key papers included data that could be directly tied to EAIS conditions at sea level. The debate, widely referred to as the ‘stabilists' versus the ‘dynamicists'^18^, became defined by these end-member solutions, with little consideration of possible intermediate ice-sheet configurations. Furthermore, none of the dozens of key papers included explicit or adequate definitions of either a ‘stable' or a ‘dynamic' ice sheet.

The Sirius Group diatoms, which include early and mid-Pliocene forms as well as some older taxa and many of indeterminate age, were initially interpreted^10^ as having accumulated as marine deposits in deep subglacial basins of the East Antarctic interior, notably the Pensacola Basin adjacent to the TAMs, during at least one Pliocene warm period. Subsequent ice-sheet advance was suggested to have eroded and transported diatomaceous sediments to the TAMs, forming Sirius Group tills. This hypothesis implied not only dramatic EAIS retreat deep into the interior of the East Antarctic subglacial basins during the Pliocene, but significant post-Pliocene mountain uplift or post-retreat ice-sheet configurations much larger than have otherwise been hypothesized. This view of a dynamic EAIS and active tectonics in the TAMs was challenged on several fronts, most notably based on evidence from landscape evolution studies^12^,^13^,^14^ and inferred mountain uplift rates^19^.

The diatom data remained a key to several aspects of the competing hypotheses, with respect to their occurrence and distribution, their identification and biostratigraphic age, and their method of emplacement. Once the occurrence of the diatoms in association with the high-altitude deposits was independently replicated^20^,^21^ and the veracity of the mid-Pliocene age of key diatoms was established^22^, it was suggested that their occurrence was not the result of subglacial transport, but of aeolian processes that significantly post-dated emplacement of the glacial facies, implying a much older (Oligocene or Miocene) age for different units of the Sirius Group. Ultimately, several studies^20^,^21^ showed that most of the diatoms, and notably those attributable to a Pliocene age, were concentrated in the outer layers of exposed tillite surfaces rather than evenly distributed throughout the diamicton, and detailed stratigraphic analysis of thick Sirius Group sediments, conducted in the Beardmore Glacier region, failed to produce age-determinant diatoms that specifically pointed to a Pliocene age^23^. This suggested that unlike *in situ* terrestrial fossils of uncertain age associated with diverse Sirius glacial facies^23^,^24^, the Pliocene age diatoms may be surficial contaminants, and therefore provide an inappropriate method for dating emplacement of the tills. Subglacial transport of the diatoms as described by Harwood and Webb^15^ may be further called into question by results of a ring-shear experiment^25^ that documented high rates of diatom fragmentation in till subjected to shear strains typical of wet-based subglacial deformation, although diatoms and diatom clasts can be transported great distances if frozen in glacier ice, whether resulting from aeolian deposition onto snow/ice surfaces or as debris frozen in basal ice.

The majority consensus that the key diatoms were emplaced by aeolian processes was widely interpreted as refutation of the ‘dynamic' EAIS hypothesis^12^,^13^,^26^. This conclusion was supported by other evidence indicating a pre-Pliocene age of most Sirius tillite surfaces, notably cosmogenic exposure dating^19^,^27^,^28^ and geomorphic evidence for stable desert landscapes, mostly above 1,000 m elevation in the McMurdo Dry Valleys region^14^,^29^. However, none of these data directly bear on the configuration of the vast EAIS during the Pliocene. The fact that most Sirius tillites likely predate the Pliocene, and that ancient surfaces may be preserved at high altitude, does not falsify the hypothesis that EAIS sectors overlying deep basins may have a history of significant retreat during Pliocene warm intervals. The latter fact is demonstrated by continued preservation of these landscapes despite extended periods of warmer than present open ocean with little sea ice in the adjacent Ross Sea during the Early Pliocene^2^, and repeated Ross Sea deglaciations through the Late Pliocene^2^ and Pleistocene^4^,^30^.

The ‘stabilist' view gained considerable support from an early generation ice-sheet model^31^ that called for an unrealistic temperature increase of +20 °C to significantly draw down the Pliocene ice sheet. In his recent review of the Sirius debate, Barrett^5^ concluded that despite compelling evidence for open marine conditions across the Ross Embayment^2^,^3^, the EAIS remained under stable, full polar desert conditions throughout the Pliocene. In support of this conclusion, he cited the modelling efforts^3^,^31^,^32^ that failed to demonstrate retreat of EAIS margins given warm Pliocene forcing. Ice-sheet retreat in these previously published ice-sheet models was driven largely by insolation and temperature-induced surface melting that required higher temperatures than have been hypothesized.

In contrast with earlier modelling efforts^3^,^31^,^32^, recent observations and modelling of ongoing^9^ and projected^33^ ice retreat along the Aurora and Wilkes basins highlight the potential susceptibility of East Antarctic outlet glaciers to rapidly retreat well beyond their current configurations. Pliocene modelling^7^,^8^,^33^,^34^,^35^ and marine drill-core records from the Wilkes Land margin^36^,^37^,^38^ provide a physical basis and geological record suggesting significant grounding line retreat into East Antarctic subglacial basins during Pliocene warm intervals. Similarly, glaciomarine deposits of the Pagodroma Group exposed along the Amery Oasis/Lambert Graben of East Antarctica document Pliocene marine incursions into East Antarctica^39^,^40^.

Here we further evaluate aeolian processes and test the hypothesis that aeolian diatoms in the TAMs were the result of Pliocene EAIS retreat, rather than evidence against ice-sheet dynamic behaviour. We evaluate coastal isostatic response over a Pliocene Antarctic ice-sheet reconstruction derived from an ice-sheet model that incorporates ice-sheet grounding zone processes, including cliff failure and hydrofracturing^7^, then simulate wind regimes under these Pliocene landscape conditions using a regional climate model (RCM)^41^ to address the long-standing debate regarding the interpretation of Pliocene-age marine diatoms recovered from Sirius Group glacial deposits in the TAMs^5^. We find that the occurrence of wind-transported Pliocene marine diatoms in the TAMs supports the view that significant sectors of the EAIS retreated during Pliocene warm intervals.

## Results

### Aeolian processes and sources

**Figure 1:**
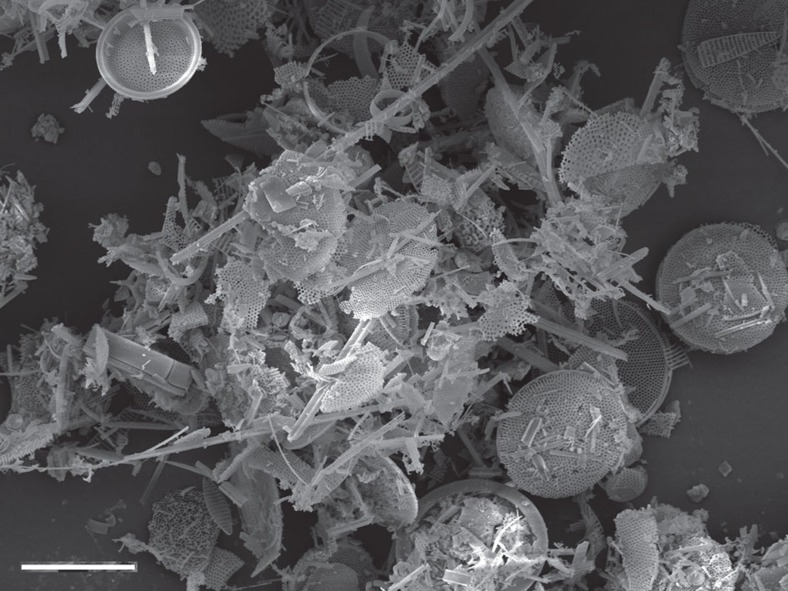
Scanning electron micrograph of diatomaceous sediment. Electron micrograph of Southern Ocean diatomaceous sediment, presented air-dried without any cleaning or processing, illustrating very high surface area of unconsolidated diatomite. Similar material is likely to have accumulated in the Wilkes and Aurora basins following retreat of ice. Once isostatically emerged and exposed they would be highly susceptible to wind erosion. Scale bar, 50 μm.

Silt and sand-sized grains, including microfossils, are well known to be raised by saltation processes and then carried significant distances by wind. Diatom frustules and small clumps of diatomaceous sediment (microclasts) have an extremely high surface-area:mass ratio (Fig. 1), making them significantly more susceptible to wind entrainment from subaerially exposed diatomaceous surfaces than mineral grains of similar size. Wind erosion and aeolian transport of diatoms typically require dry, subaerially exposed source beds—surfaces covered with abundant unconsolidated diatoms—together with appropriate surface wind regimes, including episodic storm events capable of lifting saltated particles to the mid-troposphere^42^,^43^.

Once aloft, these particles can be carried great distances, as readily seen by the observed distribution of diatoms across the Antarctic ice-sheet surface. Pleistocene and modern long-distance transport and atmospheric precipitation of diatoms on the Antarctic ice sheet and the TAMs have been demonstrated through analysis of ice cores^4^,^44^,^45^, and modern surfaces in the TAMs^46^. Aeolian diatoms in Antarctica today are strongly dominated by non-marine forms, including those characteristic of Dry Valley lake and stream deposits and some from far-flung terrestrial source beds on other continents^4^,^44^,^45^,^46^, reflecting a relative paucity of marine sources for modern aeolian dust.

The fact that marine diatoms typically dominate the diatom assemblages extracted from most Sirius tillite surfaces, despite a constant and ongoing rain of non-marine diatoms across these surfaces today, indicates that the accumulation of marine diatoms is not a recent phenomenon, but largely reflects a specific interval or intervals of significant aeolian input with distinct source beds of subaerially exposed Pliocene marine diatomaceous sediments. Diatoms are generally not lifted from ocean surfaces^47^ and carried in large numbers far from pelagic sources, owing to the weight of enclosed cytoplasm and water, as well as the consequent reduction in their surface area. This is borne out by the relative lack of modern pelagic diatoms reported from ice cores and Sirius deposit surfaces.

**Figure 2:**
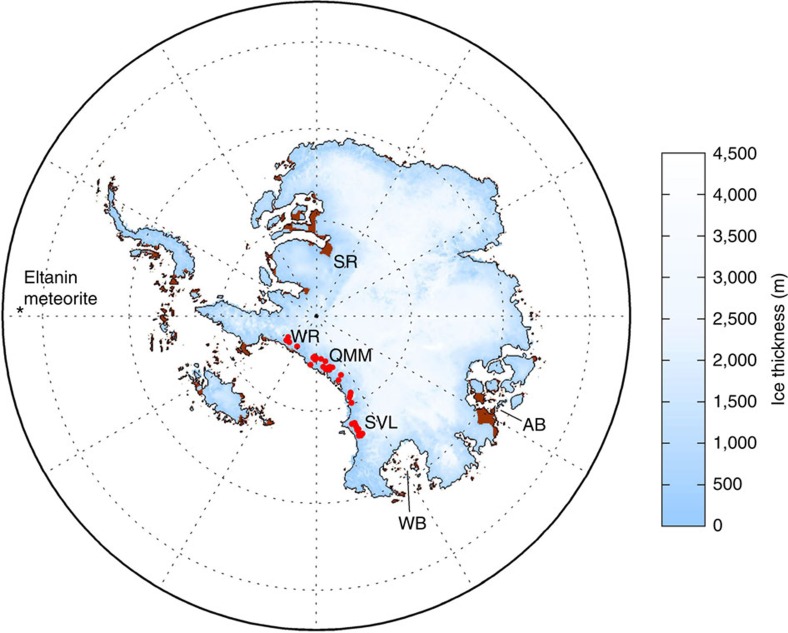
Sirius Group outcrops and Antarctic Ice Sheet configuration during warm Pliocene. Warm Pliocene Antarctic ice-sheet configuration from the simulation in Pollard *et al*.^7^, representing intervals of maximum retreat during warm austral summer orbits. Note the ice-sheet retreat across the Wilkes Basin (WB), Aurora Basin (AB) and the Recovery Ice Stream, adjacent to the Shackleton Range (SR). This model result illustrates an intermediate ice-sheet geometry, between the end-member configurations suggested in key papers in the ‘stabilist/dynamicist' debate. Coastal exposures of previously submarine deposits, following isostatic rebound (Fig. 5), are shown in brown. Diatom-bearing Sirius Group outcrops along the Transantarctic Mountains in the Wisconsin Range (WR), Queen Maud Mountains (QMM), and South Victoria Land (SVL) are indicated in red (following ref. 5). Location of the Eltanin Meteorite impact site^48^ in the Southern Ocean is indicated.

The widespread distribution of aeolian marine diatoms in Sirius deposits (Fig. 2) implies that there must have been a significant upwind source of exposed diatomaceous beds. Specific hypothesized provenance of the diatoms was rarely discussed in the papers that made the case for aeolian emplacement of Sirius diatoms, although Stroeven *et al*.^20^,^21^ noted that the East Antarctic coastal margin along the Wilkes Land coast could provide one possible source for some aeolian diatoms. Despite this acknowledgement they explicitly rejected EAIS dynamic responses to Pliocene warmth^26^. Gersonde *et al*.^48^ presented a radically different hypothesis, suggesting that the diatoms might represent ejecta from a Pliocene meteorite impact in the Southern Ocean.

### Improved models

A new version of an established Antarctic ice-sheet model (PSUICE-3D) (ref. 3), which uses hybrid ice dynamics and allows for the buttressing effects of floating ice shelves and freely migrating grounding lines, now includes physics associated with glaciological processes of melt-water enhanced calving due to hydrofracture and ice cliff failure^7^. When forced by modest atmospheric and oceanic warming representative of warm Pliocene conditions with 400 p.p.m.v. CO_2_ and a warm austral summer orbit^7^, the model produces a significant retreat of marine-based ice, including most of West Antarctica and the major Wilkes and Aurora basins in East Antarctica, where grounding lines retreat more than 500 km into the interior (Fig. 2), thus providing a physically plausible scenario for EAIS recession, with sea level rise and marine basin development. This result is further enhanced, notably in the Wilkes Basin, by the inclusion of dynamic topography^8^. Together, these new models further strengthen the case for EAIS retreat during Early and mid-Pliocene warm intervals, though do not indicate retreat as extensive as that implied by Webb *et al*.^10^. The recent modelling results are more consistent than earlier work with far-field sea-level^49^ and climate records^50^ and with East Antarctic coastal margin geologic records^37^,^38^,^39^,^40^.

### Interior seaways and rebound

The seaways that would have filled the freshly exposed, deglaciated marine basins would have been well-mixed and nutrient-rich, fed by glacier ice-derived iron and other limiting micronutrients^51^,^52^, and thus would have hosted rich planktic diatom communities, comparable to those that rapidly accumulated thick diatomaceous oozes in the Ross Embayment during some of the same episodes of Pliocene West Antarctic Ice Sheet retreat^2^.

**Figure 3:**
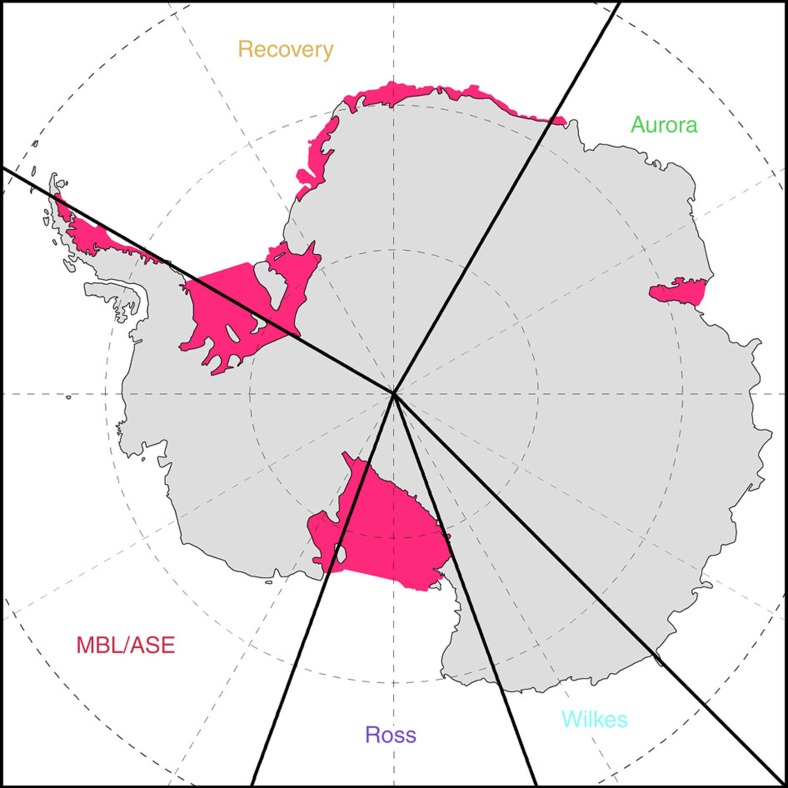
Longitudinal sectors defined to distinguish regions of ice-sheet change. Sectors are simply defined by longitude, as follows: MBL/ASE (Marie Byrd Land/Amundsen Sea Embayment) (160° W–60° W), Recovery Basin (60° W–30° E), Aurora Basin (30° E–135° E), Wilkes Basin (135° E–160° E) and Ross Basin (160° E–160° W). These are used for the model ocean and land surface calculations plotted in Figs 4 and 5. Text colours correspond with Figs 4 and 5 graphs. A separate sector was considered focusing on the Lambert Glacier/Prydz Bay area, but the model showed little net emerged coastal area, due to the prevailing steep-walled fjord-like geometry.

**Figure 4:**
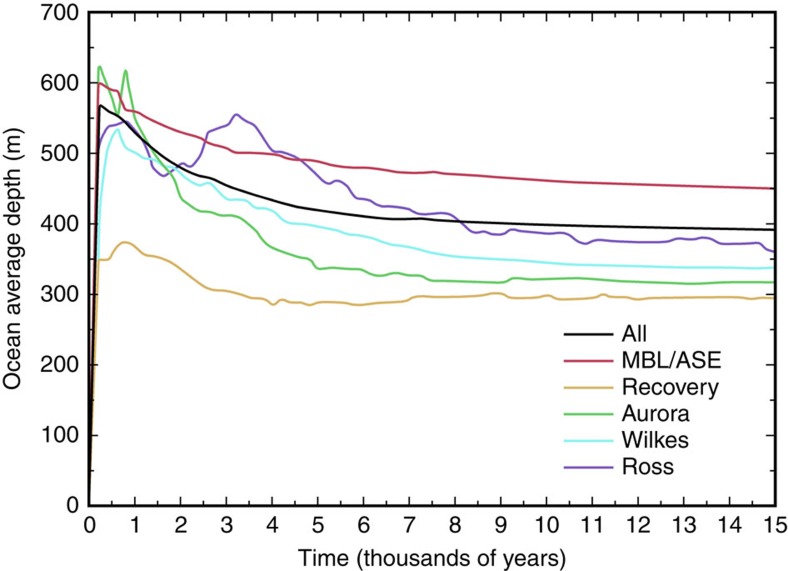
Average ocean depth change over time. Area of ‘new' open ocean versus time, that is, model cells with no ice and with bedrock depth below sea level that had been land or grounded ice at the start of the run (modern configuration), representing the new oceanic bays exposed by grounded-ice retreat. The early peak and significant subsequent decline in ocean depth in the Aurora Basin (green) and the Wilkes Basin (turquoise) reflect rapid ice-sheet collapse followed by isostatic rebound, creating expansive coastal plain and islands. Coloured lines represent sectors defined in Fig. 3.

**Figure 5:**
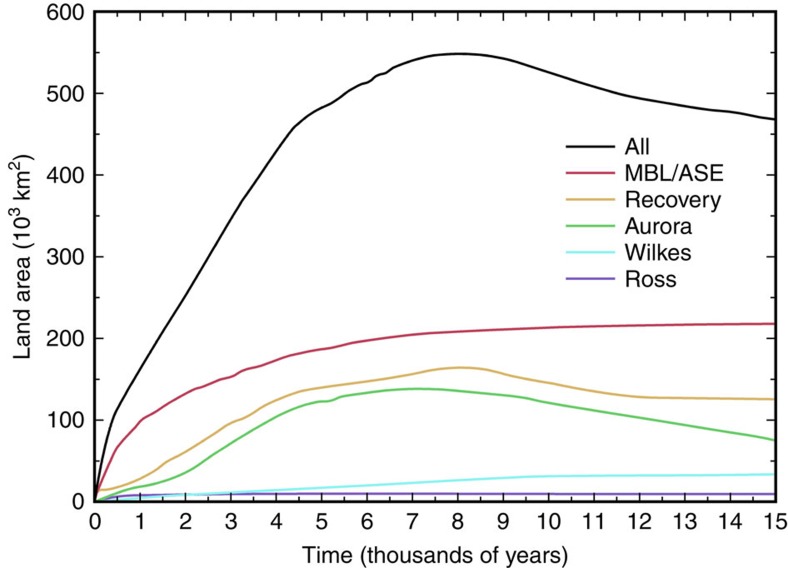
Area of exposed land over time following ice retreat and isostatic rebound. Total area of emerged exposed land (potential regions of aerially exposed diatom deposits, shown as brown patches in Fig. 2) is shown versus time through the model run, subdivided into sectors defined in Fig. 3. At each time, this is the area of model grid cells with no ice cover and with bed elevation that previously experienced deglaciation and isostatic emergence above sea level. Total for each individual sector is shown, as well as the all-Antarctic total (black curve). Note the early increase in land area of the Marie Byrd Land/Amundsen Sea Embayment sector, the late but significant rebound-induced emergence of East Antarctic basins, and the minimal emergence in the Ross Sea sector.

In the model, the lagged bedrock rebound in response to the reduced ice load becomes significant several thousand years after ice retreat, resulting in the emergence of large stretches of ice-free coastal plain and numerous islands around the East Antarctic coastline, most notably at the mouths of the Aurora and Wilkes basins, as shown in Fig. 2. Calculated basin depth and land areas are plotted by defined sectors (Fig. 3) in order to evaluate regional changes in ocean depth (Fig. 4) and emerged land area (Fig. 5) over time. According to the model, Aurora and Wilkes basins display more newly exposed land than would have emerged elsewhere around Antarctica, including the Amery Oasis of East Antarctica or around West Antarctica.

Before this emergence of ice-free land in the Aurora and Wilkes basins, the overlying ocean would have been mostly ice-free in summer, with air temperatures considerably warmer than today and little sea ice. After several millennia a significant drape of unconsolidated diatomaceous ooze would have accumulated, conformably overlaying glacial deposits. This would create a geological contact comparable to the rapid glacial-interglacial (diamicton to diatomite) transitions described in the ANDRILL-1B drillcore (defined as Motif 2)^2^.

**Figure 6:**
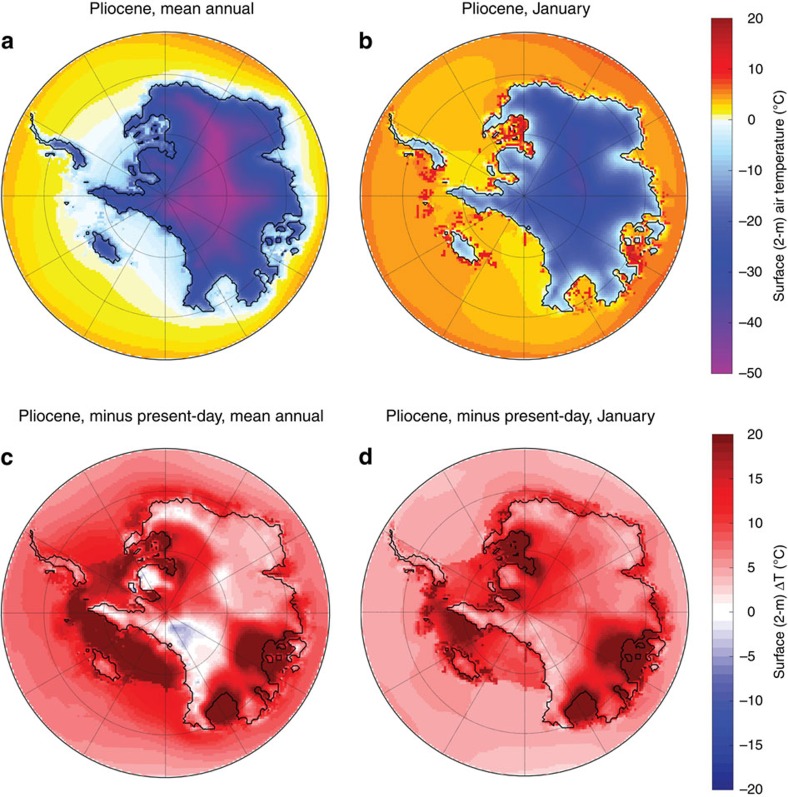
Pliocene average and summer temperatures. Pliocene calculated average annual (**a**) and summer (**b**) temperatures, and Pliocene versus modern annual (**c**) and summer (**d**) temperature anomalies. Note the temperatures much higher than modern summer values over Pliocene exposed lands of East Antarctica, indicating the probability of loss of winter snow cover over emerged Pliocene marine deposits.

Following uplift, a subaerially exposed diatom-strewn landscape would have been highly susceptible to wind erosion, especially during summer with reduced snow cover (Fig. 6), thus providing an abundant source for plumes of aeolian diatoms available to be raised aloft by storm-induced saltation. Subsequent transport across the ice-sheet surface would have led to widespread deposition on the Sirius tillite surfaces and elsewhere. Although the model shows some new land exposure in West Antarctica, an East Antarctic source for windblown diatoms is more likely, because the emerged lands around the Aurora and Wilkes sectors include extensive low-lying coastal plain and islands, whereas West Antarctic exposures are characterized by ice retreat from more steeply sloping island and mountain-front coasts, which generate less exposure of emerged coastal plain (Figs 4 and 5).

The entire process takes several thousand years following climate warming. With grounding line retreat and ice shelf collapse, a marine basin opens, with copious diatom production in open waters during summer with little sea ice. The outer basins shallow with the isostatic response following ice retreat, eventually leading to emergence of uplifted islands and coastal areas. Although the simulation in Fig. 2 shows just one episode of retreat, many such cycles of retreat and re-advance would have occurred, driven by orbital cycles on timescales of 20,000–40,000 years over a period of at least several hundred thousand years in the warm mid-Pliocene around 3 Ma ago^53^,^3^,^7^. The retreat phase of each cycle would have provided an opportunity for diatom production, with subsequent exposure and windblown transport.

### Modelling Pliocene winds

**Figure 7:**
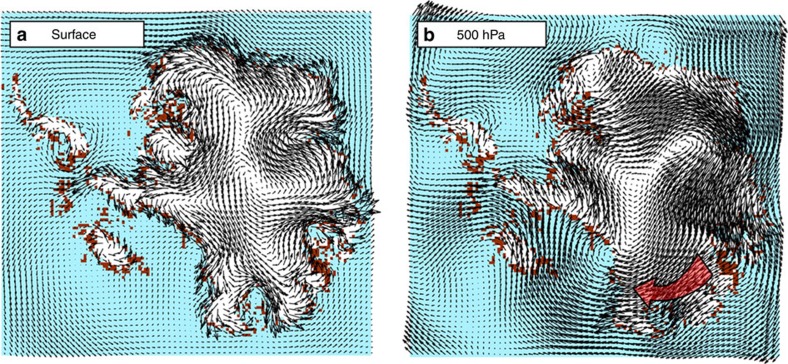
Surface and mid-troposhere winds over Pliocene Antarctic landscape. Modelled wind regimes over the warm Pliocene ice-sheet configuration (Fig. 2) during austral summer, DJF (December, January and February). (**a**) Near-surface winds are dominated by katabatic processes. Some Pliocene diatoms in the central TAMs may have been carried at relatively low altitudes from coastal exposures of a retreated Recovery Ice Stream near the Shackleton Range in the Weddell Sea sector or along the Ross Sea coast. Coastal storm events over the emerged Wilkes and Aurora basins would have provided the dominant mechanism for lifting a large volume of diatoms and other dust particles off exposed, snow-free surfaces to higher altitudes where they could be carried great distances. (**b**) Modelled winds at 500 hPa (∼5,000 m). Red arrow indicates likely dominant pathway for aeolian dust from Aurora Basin outcrops towards the TAMs. This configuration suggests that stratospheric rainout from a meteorite impact in the South Pacific Ocean would be towards the Ross Embayment, and thus ejecta would likely be more abundant along Marie Byrd Land and the Whitmore Mountains than the TAMs, though specific ejecta pathways have not investigated. Neither surface nor mid-altitude winds indicate significant pathways from the Ross Sea.

**Figure 8:**
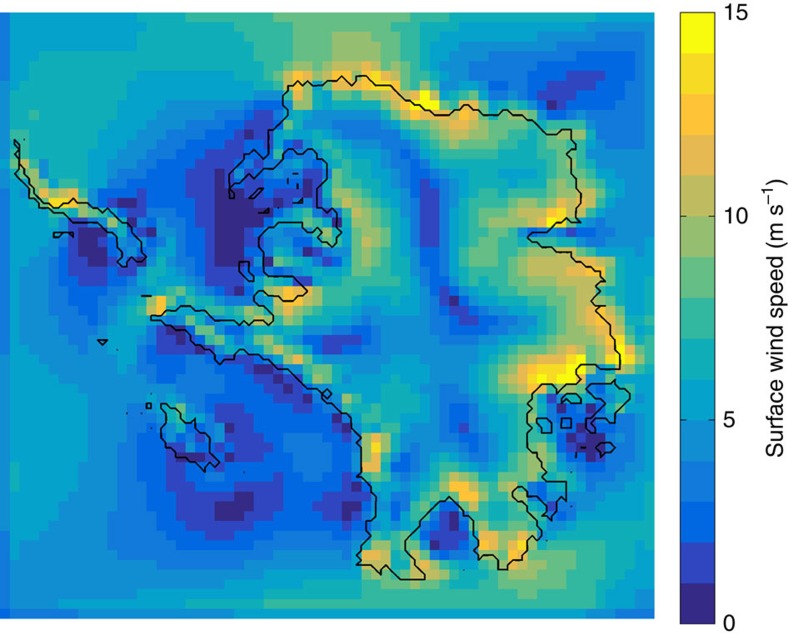
Average summer wind speed over Antarctic Pliocene landscape. Seasonally averaged summer (DJF) surface (10 m) wind speeds, corresponding to the simulated velocity vectors in Fig. 7a. Note the maximum wind speeds at the ice-sheet margin, adjacent to exposed, emergent marine basins. Antarctic summer months, when the coastal margins are snow-free under warm Pliocene conditions, may provide the best opportunity for lifting diatom tests, though storm events at any time of the year would contribute to significant wind erosion of the deposits.

To evaluate the hypothesis that marine diatoms associated with Sirius tillites were derived from aeolian transport directly resulting from EAIS partial retreat, the Pliocene wind regime is simulated using the Genesis v3 Global Climate Model with a nested, polar version of the RCM (RegCM3) over Antarctica^41^. The model simulates three-dimensional winds for a Pliocene-like climate with a retreated Antarctic Ice Sheet and East Antarctic basins with exposed subaerial land as described above. The near-surface winds are mainly katabatic and do not show direct transport from either the Wilkes or Aurora basin surface exposures towards the Sirius locations in the TAMs (Fig. 7a). Surface winds do, however, suggest a potential for some aeolian transport towards the central TAMs from the Recovery Basin sector coast, adjacent to the Shackleton Range (Fig. 2). We consider the Ross Sea sector as a relatively insignificant source of windblown diatoms because there is little isostatic uplift compared with the Aurora and Recovery sectors. Like today, the modelled Pliocene low-level winds on the fringes of the ice sheet are energetic, with summer (December, January and February) average wind speeds exceeding 10 m s^−1^ in many locations adjacent to the freshly exposed sediments, most notably along the Aurora Basin coastline (Fig. 8). Wind speeds during coastal storm events would have been significantly higher, with low-pressure events enhancing the process of lifting diatoms to the mid-troposphere, where long-distance transport is possible. We suggest that the aeolian process was enhanced during austral summer, because modelled surface temperatures (Fig. 6) indicate complete loss of winter snow cover over emerged land in East Antarctica.

In contrast with surface winds, mid-tropospheric winds at 500 hPa, ∼5,000 m altitude, are generally cyclonic over the continental interior and show strongly favourable transport directions towards the TAMs and Sirius outcrop localities, from both Wilkes and Aurora basin marine sediment exposures (Fig. 7b), especially during austral summer.

Although ice-free bedrock patches also emerge around the islands of West Antarctica (Fig. 2), both surface and upper-level winds are unfavourable, blowing away from the TAMs (Fig. 7), which implies they were not a significant source of particulates on Sirius Group deposits. Previous studies^39^,^54^ have focused on geological evidence of Pliocene and earlier retreat in the Lambert basin. Our model shows some retreat there (Fig. 2), in basic agreement with ice-erosion modelling of Taylor *et al*.^55^ and Jamieson *et al*.^56^. However the extent of retreat is much less than in the Wilkes and Aurora basins, and there is significantly less exposed ice-free land available to provide large volumes of aeolian diatoms (Fig. 2).

## Discussion

Despite a paucity of exposed source beds today, there is widespread evidence for rare diatoms from coastal and Dry Valley sites being raised to the Polar Plateau, but these are dominantly of non-marine origin^4^,^44^,^45^,^46^, rather than marine. There is also long-distance aeolian transport of mineral dust^57^,^58^ and diatoms over Antarctica. Our analysis supports the view that Pliocene diatoms sourced from freshly exposed marine sediment in the deglaciated East Antarctic basins were lifted, in large numbers, to mid-level elevations by energetic low-level Antarctic winds and transported across the ice sheet towards the TAMs; these processes remain in effect today, but from far more limited source beds. We assert that this mechanism provides a more satisfactory explanation for the Sirius diatom data than the Eltanin meteorite hypothesis^48^, which would imply a more widespread and uniform distribution of the diatoms than is seen. However, we note that this impact hypothesis cannot be disproven, because there has not been a concerted effort to either model the ejecta volume and aerosol pathways, or search for this distinctive airfall outside of the TAMs region, such as along the Whitmore Mountains or Antarctic Peninsula, closer to the source. Pollard *et al*.^7^ show Pliocene EAIS retreat and DeConto and Pollard^33^, using the same model, forecast similar retreat and sea level rise in future centuries, but their model shows no significant EAIS retreat during late Pleistocene interglacials, which may explain the apparent absence of distinctly Pleistocene age diatoms in Sirius deposits.

Accurate Pliocene meteorological and ice-sheet reconstructions become more relevant as new modelling indications of current and potential future^33^ EAIS instability come to light, especially given that modern atmospheric CO_2_ has now reached Pliocene levels and is expected to continue to rise. Scientific debates, such as the interpretation of Sirius Group diatom data, often turn on key details that if misinterpreted or interpreted through a rhetorical bias may obscure broader truths. The debate between ‘stabilists' and ‘dynamicists' focused on the configuration of the EAIS during the Pliocene, but none of the published studies presented data that was directly related to or indicative of EAIS geometry or thickness during the Pliocene. Furthermore, neither a ‘stable' nor ‘dynamic' ice sheet was adequately defined in context, and intermediate ice-sheet configurations, such as those suggested by new models, were not considered. The IODP Sites 1165 (ref. 37) and U1358 (ref. 38) and ANDRILL-1B (ref. 2) drillcores provide previously unavailable information regarding environmental conditions in Antarctica's marginal seas during Pliocene warm intervals. These and other more distal records^50^ point to a highly variable Pliocene climate, including intervals significantly warmer than present with high sea-levels^49^, consistent with retreat of the ice margin into deep subglacial basins. This configuration is defendable given the 10–20 m sea-level uncertainty owing to dynamic topography and glacial isostatic adjustment distortions of Pliocene shoreline mapping^49^.

The new model presented here implies significant EAIS retreat during the Pliocene with wind patterns that can explain the source and mechanism for emplacement of Pliocene marine diatoms in the TAMs by aeolian processes. Webb *et al*.^10^ initiated an important discussion regarding dynamic behaviour of the EAIS during the Pliocene. Although their interpretation of a glacial origin for the diatoms in the Sirius tillites and the extent of retreat that they inferred is not supported, we suggest that these Pliocene marine diatoms nevertheless provide evidence of significant EAIS retreat from the current coastline during Pliocene warm intervals—enough to have had a significant global impact on sea level. When this debate began, decades ago, it was already understood that constraining past ice-sheet dynamics is important for forecasting future behaviour in a warming world.

## Methods

### Ice-sheet area model

The retreated Pliocene Antarctic ice-sheet configuration is obtained from a simulation with an established three-dimensional ice-sheet model^7^, driven by climatic conditions representative of the warm mid-Pliocene and with a warm austral summer orbit representing Antarctic interglacial conditions. The model includes floating ice shelves and grounding line migration, and uses hybrid ice dynamics with an internal condition on ice velocity at the grounding line, with a grid size of 10 km. Bedrock deformation is modelled as an elastic lithospheric plate above local isostatic relaxation. Two additional mechanisms^7^ are added to enable grounding-line retreat into East Antarctic subglacial basins: (i) hydrofracturing and loss of buttressing by floating ice shelves due to surface melt; and (ii) structural failure of ice cliffs at the grounding line.

### Sector definition

To help distinguish relative land area exposed (Fig. 4) with ice retreat and subsequent rebound (Fig. 5) we separated the Antarctic landmass and marginal seas into five sectors (Fig. 3): Wilkes Basin (135° E-160° E), Ross Basin (160° E-160° W), Marie Byrd Land/Amundsen Sea Embayment (160° W-60° W), Recovery Basin (60° W-30° E), and Aurora Basin, which includes the Prydz Bay/Lambert Graben area (30° E-135° E). The Lambert Glacier region was folded into the Aurora Basin sector because it shows little significant change with ice retreat, compared with that of the Aurora Basin region. This is because steep slopes along the expanded Lambert Valley limit exposure of broad emerged coastal plain deposits relative to the Aurora area.

### Atmospheric modelling

To drive the ice model, surface mass balance was computed from decadal-average monthly temperature and precipitation of the RegCM3 RCM^41^ with small modifications for polar regions, run over a domain spanning Antarctica at 40 km horizontal resolution^7^ and with 18 vertical levels. For this RegCM3 run, the ice sheet was specified at its modern state, except that all West Antarctic marine ice is replaced by ocean, and given meteorological lateral boundary conditions provided by the GENESIS v3 Global Climate Model. Atmospheric CO_2_ was set to 400 p.p.m.v. representative of the mid-Pliocene warm interval, and an orbit common to the mid-Pliocene that yields particularly intense austral summer insolation was used. Prescribed greenhouse gas concentrations and astronomical calculations of insolation are identical in the GCM and nested RCM. For the ice-sheet's parameterization of sub-ice-shelf oceanic melting, a uniform increase of +2 °C was added to a modern ocean climatology, consistent with conservative Pliocene paleoceanographic reconstructions^2^,^50^.

Pliocene mean annual and summer (January) temperatures are calculated from the RCM (Fig. 6). These highlight temperature anomalies of up to 20 °C relative to today over the emerged land areas of Aurora and Wilkes basins. Wind patterns in Fig. 8 are decadal averages of austral summer (December, January and February) from an additional RCM simulation of warm-Pliocene conditions as above, but with the Antarctic Ice Sheet configuration reset to the retreated Pliocene state shown in Fig. 2. In this case, surface boundary conditions in the RCM are provided by the 10 km ice-sheet model, interpolated to the 181 × 181, 40 km polarstereographic RCM grid centred on the South Pole. The RCM grid extends sufficiently equatorward of the Antarctic margin to allow a generous buffer between Antarctica and the nested GCM-RCM boundaries. Winds representative of 500 hPa heights are from the model's eighth vertical level (*σ*=0.5100). Surface winds are from the lowest model level (*σ*=0.9950).

Plots for both the ice sheet and atmospheric model outputs were prepared using MATLAB software (MATLAB and Mapping Toolbox Release 2015b, The MathWorks, Inc., Natick, Massachusetts, United States).

### Data availability

Ice-sheet and RCM model outputs are available on request. Netcdf-format output file is available for the ice-sheet distribution in Fig. 2, which is derived from the main simulation in ref. 7. Tables are available on request for the time series in Figs 4 and 5.

## Additional information

**How to cite this article:** Scherer, R. P. *et al*. Windblown Pliocene diatoms and East Antarctic ice sheet retreat. *Nat. Commun.* **7,** 12957 doi: 10.1038/ncomms12957 (2016).
